# Determination of Kanamycin by High Performance Liquid Chromatography

**DOI:** 10.3390/molecules24101902

**Published:** 2019-05-17

**Authors:** Xingping Zhang, Jiujun Wang, Qinghua Wu, Li Li, Yun Wang, Hualin Yang

**Affiliations:** 1 College of Life Science, Yangtze University, Jingzhou 434025, China; xpzhang1987@163.com (X.Z.); wangjiujun2018@126.com (J.W.); wqh212@hotmail.com (Q.W.); lily2012@yangtzeu.edu.cn (L.L.); 1wangyun@yangtzeu.edu.cn (Y.W.); 2 Research and Development Sharing Platform of Hubei Province for Freshwater Product Quality and Safety, Yangtze University, Jingzhou 434025, China

**Keywords:** Kanamycin, HPLC, sample pre-treatment, different detectors, food contamination

## Abstract

Kanamycin is an aminoglycoside antibiotic widely used in treating animal diseases caused by Gram-negative and Gram-positive infections. Kanamycin has a relatively narrow therapeutic index, and can accumulate in the human body through the food chain. The abuse of kanamycin can have serious side-effects. Therefore, it was necessary to develop a sensitive and selective analysis method to detect kanamycin residue in food to ensure public health. There are many analytical methods to determine kanamycin concentration, among which high performance liquid chromatography (HPLC) is a common and practical tool. This paper presents a review of the application of HPLC analysis of kanamycin in different sample matrices. The different detectors coupled with HPLC, including Ultraviolet (UV)/Fluorescence, Evaporative Light Scattering Detector (ELSD)/Pulsed Electrochemical Detection (PED), and Mass Spectrometry, are discussed. Meanwhile, the strengths and weaknesses of each method are compared. The pre-treatment methods of food samples, including protein precipitation, liquid-liquid extraction (LLE), and solid-phase extraction (SPE) are also summarized in this paper.

## 1. Introduction

Kanamycin is widely used in the treatment of animal infections, added as growth promoters or feed additives for preventive therapy [1]. The antibacterial mechanism of kanamycin is that it can irreversibly bind to the bacterial ribosomal 30S subunit and inhibit its protein synthesis [2]. Because of its potential ototoxicity and nephrotoxicity [3,4,5,6], the indiscriminate use of kanamycin will enhance bacterial resistance and cause kanamycin-residue accumulation in animal-derived food, which threatens human health. Therefore, the European Union has promulgated regulations on the maximum residue limits (MRLs) of kanamycin in different food matrices (100 μg/kg for muscle, 100 μg/kg for egg, 600 μg/kg for liver, 2500 μg/kg for kidney, 150 μg/kg for milk and 100 μg/kg for chicken meat) [7].

**Figure 1:**
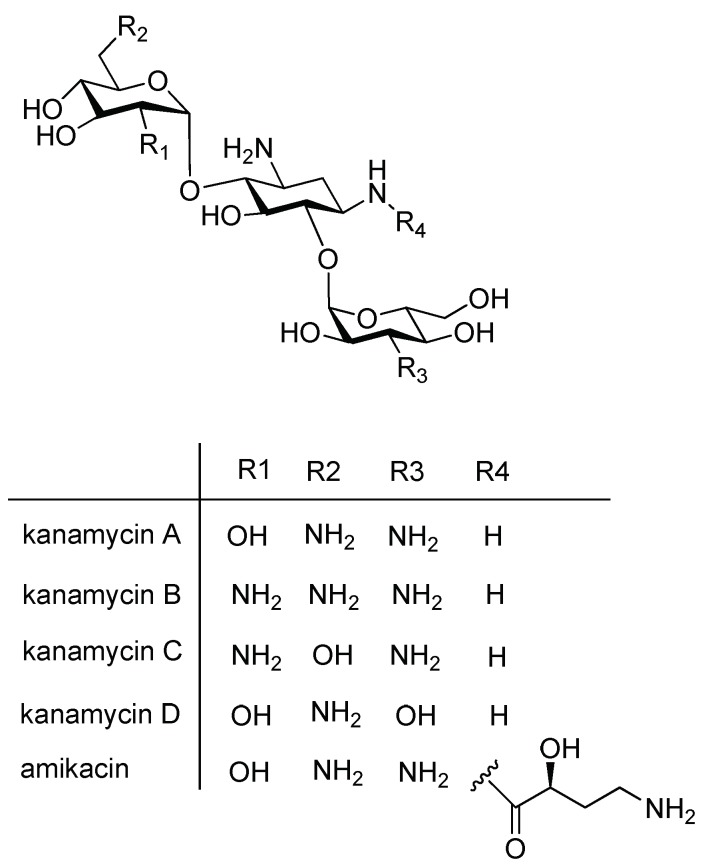
Structure of kanamycin A, B, C, and D and amikacin.

Kanamycin was isolated in 1957 [8]. It is a mixture of several closely related compounds, such as main constituent kanamycin A (>95%), as well as minor constituents kanamycin B, C, and D (<5%). The major components are shown in Figure 1 [9]. In addition, degradation products such as 2-deoxystreptamine and paromamine can also be present [10]. Kanamycin A and C are isomers, whereas kanamycin B and D have different functional groups [9].

## 2. The Pre-Treatment Methods of Food Sample

The key point of detecting kanamycin is to remove the impurities or extract kanamycin from matrices. The usual techniques for extraction and cleanup of kanamycin from matrices include protein precipitation, liquid-liquid extraction (LLE), and solid-phase extraction (SPE) [11]. Based on these techniques, pre-treatment methods for kanamycin detection in food samples are summarized as follows.

### 2.1. Protein Precipitation

Deproteinization was commonly used in the extraction of kanamycin from biological matrices because removal of interferences is necessary to retain good recoveries. Acetonitrile, acidified methanol, and trichloroacetic acid were commonly used precipitation reagents.

In human plasma sample, the simple organic solvent of acetonitrile was used for deproteinization with kanamycin recovery range from 92.3% to 100.8% [12,13]. The acidified methanol with a final concentration of 0.13 mol/L hydrochloric acid (HCl) can also be used for deproteinization of human plasma, and kanamycin recovery ranges from 91.2% to 93.4% [14].

In rat plasma samples, trichloroacetic acid (TCA) with a final concentration of 25–30% was a good precipitation reagent and offers best recovery [15]. 

In human serum sample, the acidified methonal with a final concentration of 0.14 mol/L HCl can be used to extract kanamycin [16]. Meanwhile, TCA with a final concentration of 40% can be applied in human serum deproteinization, and recovery of kanamycin ranges from 93.9% to 98.4% [17]. 

Dried blood spots (DBSs) were more convenient than traditional venous blood sampling. In one anti-TB drug analysis, 0.1 mol/L HCl in mixed methonal solution was used for deproteinization of DBS samples [18]. 

Pig feeds samples were extracted with 0.1 mol/L HCl and kanamycin recovery ranged from 89.4% to 92.8% [19].

In bovine milk, swine and poultry muscle, samples were first precipitated by 15% TCA and then purified with bulk C18 resin. The recoveries of kanamycin were 92% in milk and 36.8–67% in muscle [20].

The chicken meat samples were extracted and precipitated with a mixture of acetonitrile (ACN)-2% TCA (45:55, *v*/*v*), followed by on-line clean-up using turbulent flow chromatography [21]. This automated on-line technique enabled a larger number of samples to be analyzed per day than the traditional clean-up technique. Kanamycin recovery ranged from 109% to 120% in chicken meat [21].

### 2.2. Liquid-Liquid Extraction

Liquid-liquid extraction (LLE) has been exploited as an extraction procedure for kanamycin from complex matrices. In a published method, veal muscle samples were extracted using CH_3_CN-H_2_O (86:14 *v*/*v*), followed by a defatting step using hexane liquid-liquid extraction [22].

A new pre-preparation technique of dispersive liquid-liquid microextraction based on solidification of floating organic droplet (DLLME-SFO) was developed, which is a new kind of LLE method that could be applied in the analysis of volatile and polar compounds, like kanamycin. In wastewater and soil, kanamycin is extracted with dodecanol (extraction solvent) and ethanol (dispersive solvent) [23]. Compared with conventional sample preparation methods, the proposed derivatization followed by DLLME-SFO procedure significantly reduced the consumption of organic solvent with high enrichment factor. The DLLME-SFO method facilitated high extraction efficiency and further wide linear range, with good precision, and lower detection limit. The recovery was found to be between 91.3–102.7% for wastewater and 90.3–107.7% for soil. The linearity range was 0.5–500 ng/mL. The LOD was 0.012 ng/mL and LOQ was 0.05 ng/mL [23].

### 2.3. Solid-Phase Extraction

In many cases, solid-phase extraction (SPE) have been extensively used to extract and concentrate trace organic materials from samples [24,25,26]. According to packing materials, the solid phase extraction can be classified into four types: Bonded silica gel particle, high polymer material, adsorptive packing material, and mix-mode and specialized column. In this review, the sorbents used for kanamycin analysis mostly belong to the bonded silica gel type, except for molecularly imprinted polymers (MIPs) [27,28] and Chromabond HR-XC [29], which are a high polymer type sorbent.

**Figure 2:**
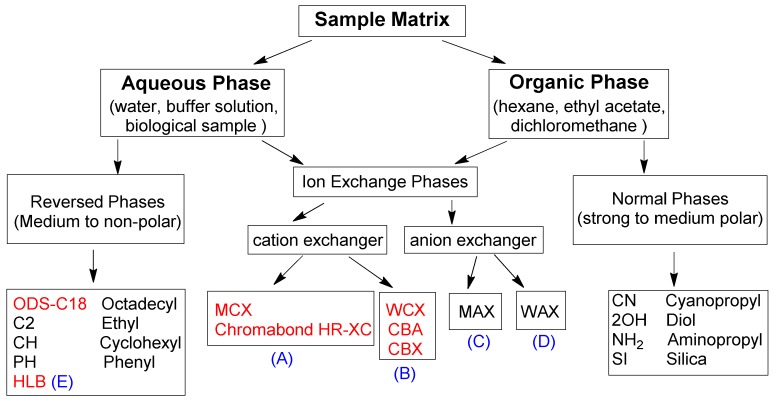
The classification and choice of solid-phase sorbents. (A) Strong cation exchanger; (B) Weak cation exchanger; (C) Strong anion exchanger; (D) Weak anion exchanger; (E) Hydrophilic-lipophilic balanced co-polymer-reversed phased retention.

According to different retention mechanisms, the SPE sorbents used in this review could be further classified into reversed phases sorbents, ion exchange phases sorbents (cation exchanger and anion exchanger), and normal phases sorbents, as shown in Figure 2. The SPE sorbents included in this review are reversed phase sorbents (ODS-C18, Sep-pak tC18, Oasis HLB), strong cation exchanger (Oasis MCX, Chromabond HR-XC), and weak cation exchanger (WCX, CBA, CBX). 

The reversed phase sorbent Sep-pak tC18 [30] and ODS-C18 [31] was able to extract the non-polar compound from the aqueous sample. The porous silica particles surface bonded with C18 or other hydrophobic alkyl groups. Because of its hydrophobic distribution mechanism, it has strong retention with hydrophobic compounds, but weak retention with hydrophilic compounds. Before use, the cartridge must be preconditioned with a water-soluble organic solvent to solvate the alkyl chains, and then equilibrated with water. It must then be loaded with aqueous samples, followed by eluting with water. A drawback is that before loading the sample, the sorbent must be kept wet, otherwise it will result in low analyte recovery or poor reproducibility. The AccuBOND ODS-C18 cartridge was used for cleanup in soil samples with a kanamycin recovery range from 72.3% to 92.5% [31].

The HLB cartridge has both hydrophilic and lipophilic functional groups, which is a new hydrophilic-lipophilic balanced wettable reversed-phase sorbent [32]. It can overcome the limitations of traditional reversed phase sorbents. Firstly, it is wettable with water, so it has good recovery and reproducibility even the cartridge runs dry during processing. Secondly, it is available for a wide range of compounds including both polar and non-polar chemicals. In muscle, kidney, liver, honey and milk samples, kanamycin was extracted through two consecutive Oasis HLB cartridges (3 mL/60 mg) with a recovery range from 71% to 104% [33].

Ion exchange sorbents (MCX, WCX, MAX, WAX) were found to extract ionizable compounds from the aqueous sample. Because of the ion exchange and hydrophobic distribution mechanisms, the ion exchange sorbents have a strong retention to ionic compounds that have the opposite electric charge of the sorbent carrier, but very weak retention to other compounds [34].

The MCX cartridge is a mixed-mode reversed-phase strong cation exchanger with a pKa of less than 1.0; its sulfonic acid groups have high selectivity to alkaline compounds. Prior to use, it was preconditioned with MeOH, followed by water, then loaded with the extracted sample. Kanamycin is a weak alkaline compound with a pKa of 7.2. At pH lower than 5, the kanamycin was essentially charged and absorbed in the cation cartridge; thus, the sample was extracted with strong acid of 0.1 mol/L HCl [35], 10% TCA [36] or 9% FA [37] aqueous solution prior to loading. At pH higher than 9.0, the kanamycin was neutralised, and the elution could take place. Thus, ammonium methanol solution (1–25%, pH 9.2) was applied to elute kanamycin from the sorbent.

The MCX cartridge was used to extract samples in animal feeds [35], swine tissue [36] and human serum [37] with a kanamycin recovery of 98.4–106% [35] and 80.7% to 91.3% [36], respectively.

The Chromabond HR-X cartridge was styrene-divinylbenzene copolymer based strong cation exchanger. Its surface bonded to benzenesulfonic acid groups [38]. Thus, its retention mechanism was similar to the Oasis MCX sorbent. It was used for cleanup in muscle, kidney and milk samples, with kanamycin recovery ranging from 95% to 102% [29].

The WCX cartridge is a mixed-mode reversed-phase weak cation exchanger with pKa of about 5.0. Its carboxyl groups have high selectivity to strong alkaline compounds. Prior to use, it was preconditioned with MeOH, followed by water, then loaded with the extracted sample. At pH over 6.5, this sorbent was essentially charged to retain kanamycin, so the PH of extracted sample was adjusted to 6.5~7.5 with NaOH and HCl prior to loading. At pH lower than 3.0, the charge on the sorbent was neutralised, and the elution could take place. So, ammonium formate buffer solution (pH 3) [39,40,41] orformic acid 10% [42], 40% methanol solution [30] were applied to elute kanamycin from the sorbent.

The WCX cartridge (Accell plus CM) was used for cleanup in honey and kidney samples, with kanamycin recovery range from 82% to 105% [40]. The Taurus WCX cartridge was used in honey, milk and liver samples, with a kanamycin recovery range from 58% to 96% [41]. Consecutive SPE cleanup using Sep-pak tC18 and Oasis WCX were applied in milk sample, with a reduced matrix effect and improved absolute kanamycin recoveries from 69.9% to 77.9% [30]. Lehotay et al. used DPX SPE (conducted in a pipet tip rather than a cartridge or centrifuge tube) with 5 mL tips (10 per row) containing 50 mg WCX sorbent for the cleanup of bovine kidney, liver, and muscle samples. The recovery of kanamycin was from 82% to 94% at a spiking level of 0.1 μg/g [42].

The carboxylic acid (CBA) cartridge was a weak cation exchanger with pKa of about 4.8, similar to the Oasis WCX cartridge. Ammonium acetate (pH 7.0) was chosen as the conditioning solution. The pH of the extracts was adjusted to pH 7.5. The 2% FA in methanol was applied to elute kanamycin from the sorbent. It was used to purify the extracts in muscle, liver, kidney, milk and egg samples [43].

The carboxypropyl (CBX) cartridge was a weak cation exchanger, similar to the Oasis WCX cartridge. The pH of the tissue extract was adjusted to pH 7.0, and then passed slowly through the CBX column. The column was washed with water and then eluted with 5 mL of acetic acid-water-methanolmixture (1:1:8) to get kanamycin; final recoveries range from 81.1% to 104% [44].

Recently, novel sorbents such as molecularly imprinted polymers (MIPs) have emerged [28,45,46]; they are synthetic materials that provide complementary binding sites to specifically capture the target analyte kanamycin. Thus, they are ideal for selective extraction and to reduce the matrix effect. MISPE-Aminoglycoside cartridges (50 mg, 3 mL) were used for extraction and clean-up processes for honey, pork and milk samples, achieving kanamycin recoveries within 78.2–97% and 70–106%, respectively [27,28]. The matrix effect results were both lower than 15%, showing that this method provided very clean extracts [27,28].

## 3. Liquid Chromatography Methods

HPLC is a conventional analytical method because of its low demand for instruments, and has been widely used in the analysis of kanamycin in different samples [36]. Depending on the retention mechanisms, the chromatographic columns used in this review were mainly divided into three types: Reversed-phase (RP) column, mixed-mode column, and hydrophilic interaction chromatography (HILIC) column. Each column type is marked in Table 1, Table 2 and Table 3. The different detectors coupled with HPLC mainly include UV/Fluorescence, Evaporative Light Scattering Detector (ELSD)/Pulsed Electrochemical Detection (PED), and Mass Spectrometry. The following content will be unfolded mainly on the basis of the classification above.

### 3.1. UV and Fluorescence-Reserved Phase Liquid Chromatography after Derivatization

Kanamycin is very polar and lacks chromophore or fluorophore, which makes it difficult to separate using traditionally reverse phase liquid chromatography (RPLC) recruiting UV or fluorescence monitoring. To overcome this problem, researchers have employed many different pre-column or post-column derivatization agents [47]. 

Derivatization of kanamycin is mainly focused on modifying its primary amine functions. The commonly used pre-column derivatization reagents include Phenylisocyanate (PIC) [48], 4-chloro-3,5-dinitrobenzotrifluoride(CNBF) [31], 1-naphthyl isothiocyanate (NITC) [13] and 9-fluorenylmethyl chloroformate (FMOC-Cl) [23]. Another reagent *o*-phthaldialdehyde (OPA) [35] can also be employed both in pre-column and post-column derivatization. Table 1 shows HPLC applications in the analysis of kanamycin with UV and fluorescence detection.

#### 3.1.1. Pre-Column Derivatization

**Figure 3:**
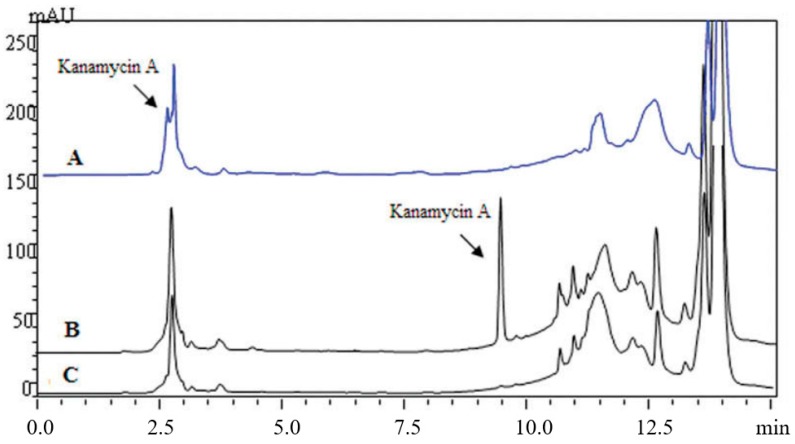
The HPLC-UV Chromatogram of CNBF-kanamycin A derivative. (A) The retention time of CNBF-kanamycin A derivative was 2.71 min without TFA in the mobile phase. The derivative could not be separated completely with interference. (B) The 0.1% TFA could improve separation efficiency. A perfect separation of CNBF-kanamycin A derivative was obtained with retention time of 9.58 min. (C) Blank soil sample.

Pre-column derivatization of kanamycin changes its polarity, which optimizes its applicability for being analyzed through conventional RPLC. For example, CNBF was used as a pre-column derivatization reagent in kanamycin analysis in different kinds of soil samples with a UV detector at 245 nm with the reaction scheme as presented in Table 4 [31]. CNBF was able to react with primary and secondary amines in alkali condition, producing stable N-substituted-2, 6-dinitro-4-(trifluoromethyl)-benzamine derivative [49]. Unlike FOMC-Cl, CNBF does not need to be removed after derivatization. The analytical column was a kromasil C18 ODS column (250 × 4.6 mm, 5 μm). The SPE column was an AccuBOND ODS-C18 (3 mL/200 mg). Linearity range was 0.01–10.0 mg/kg, and LOD was 0.006 mg/kg. The HPLC-UV Chromatogram of CNBF-kanamycin A derivative is shown in Figure 3 [31].

**Figure 4:**
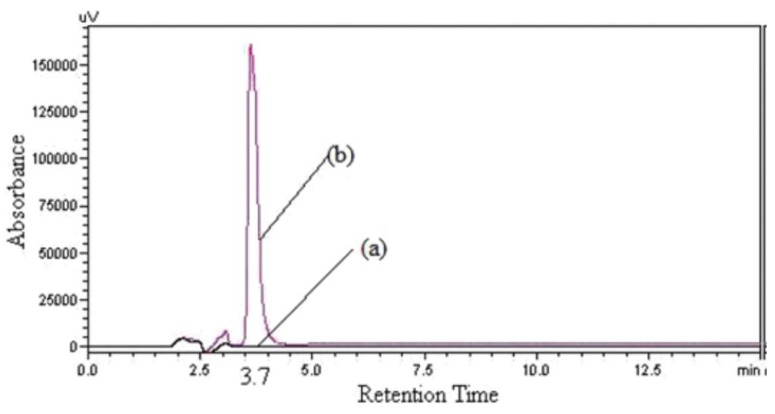
The HPLC-UV Chromatogram of the kanamycin-PIC derivative. (a) Blank; (b) Kanamycin-PIC derivative, 10 mg/mL showing retention time at 8.5 min.

PIC could react easily with primary or secondary amines, forming the stable *N*-aryl-*N*′-phenyl urea derivative, which was detected by UV at 242 nm. In Patel’s study, a corresponding derivative through reaction of KANA with PIC (5 mg/mL in ACN) was formed in the presence of TEA for 10 min, followed by the RPLC method. The derivatives were separated on a Phenomenex C18 column (250 × 4.6 mm, 5 μm). Linearity range was 5–15 μg/mL. LOD was 0.597 μg/mL. The reaction scheme of PIC with kanamycin is presented in Table 4. The HPLC-UV Chromatogram of the kanamycin-PIC derivative is shown in Figure 4 [48]. 

**Figure 5:**
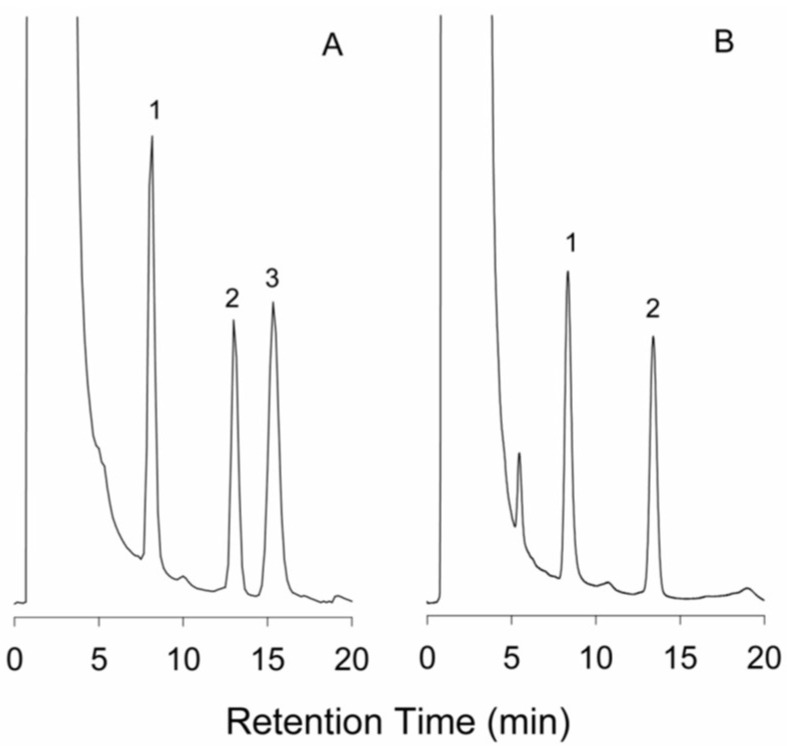
HPLC-UV chromatogram of the kanamycin-NITC derivative. (A) Separation of kanamycin A from kanamycin B, each at 40 μg/mL; (B) Determination of kanamycin A in commercial capsule sample. Peaks: 1, kanamycin A-NITC derivative; 2, acenaphthene (IS), 3, kanamycin B-NITC derivative.

NITC was used as a pre-column derivatization reagent to detect kanamycin A in human plasma by UV at 230 nm. The mixture containing kanamycin A was reacted in pyridine for 1 h. Methylamine was added to eliminate the remnant NITC after derivatization. The stationary phase was a Purospher STAR RP-18 column (55 × 4 mm, 3 μm). Linearity range was 1.2–40 μg/mL, and LOD was 0.3 μg/mL. The reaction scheme of NITC with kanamycin is presented in Table 4. The HPLC-UV Chromatogram of the kanamycin-NITC derivative is shown in Figure 5 [13].

**Figure 6:**
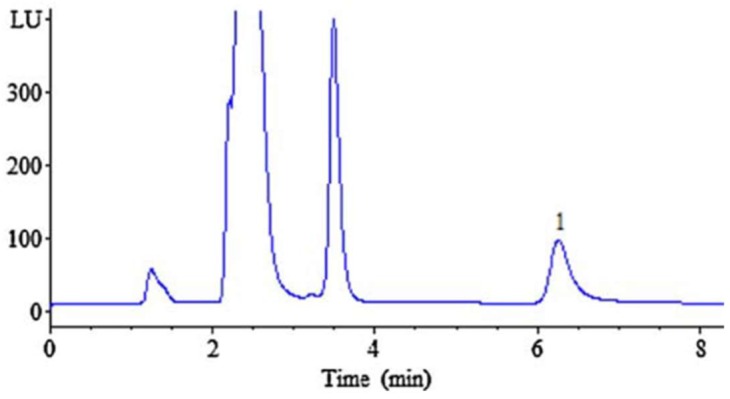
The HPLC-FL Chromatogram of the kanamycin-FMOC derivative. Kanamycin extracted from plasma from the same person 1.5 h after oral administration of 0.75 g of the drug. Peak 1, kanamycin-FMOC derivative.

FMOC-Cl was commonly used as a pre-column derivatization reagent of kanamycin, and the following detection was conducted by fluorescence. Kanamycin in human plasma reacted with FMOC-Cl in borate buffer solution (pH 8.5) for 30 min at room temperature, then separated by an Eclipse XDB C8 column (150 × 4.6 mm, 5 μm). LOD was 0.01 μg/mL, fluorescence wavelength was set at excitation of 268 nm and emission 318 nm. The reaction mechanism is shown in Table 4. The HPLC-FL Chromatogram of the kanamycin-FMOC derivative is shown in Figure 6 [12]. Similarly, pre-column FMOC-Cl derivatization of kanamycin was performed in swine tissue. The sample tissue was purified with the MCX SPE column. The derivatives were separated on a Waters symmetry C18 column (150 × 3.9 mm, 5 μm). LOD was 0.03 mg/kg for muscle, 0.06 mg/kg for liver and 0.18 mg/kg for kidney. The fluorescence measurements were set as excitation wavelength at 260 nm and emission wavelength at 315 nm. LOQ was 0.025 μg/mL, which was far lower than that reported by other researchers [36]. Another FMOC-Cl derivatization was prepared in wastewater and soil using a Diamonsil C18 column (250 × 4.6 mm, 5 μm). This is the first reported analysis that reduced the kanamycin derivative with the DLLME-SFO procedure. The fluorescence was measured at excitation wavelength 265 nm and emission wavelength 315 nm [23].

**Figure 7:**
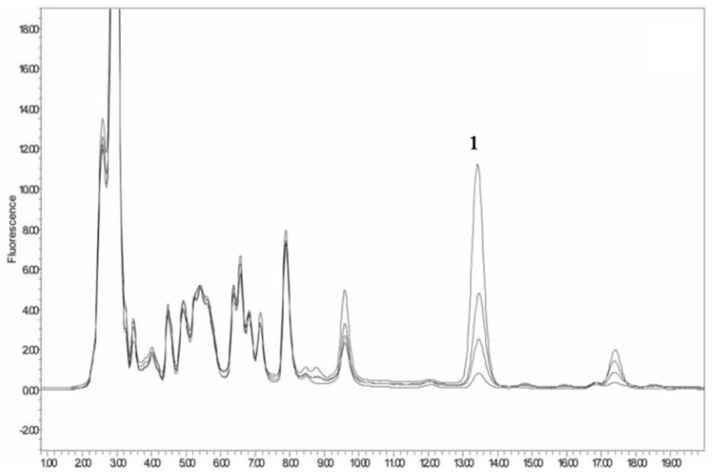
The HPLC-FL Chromatogram of kanamycin-OPA pre-column derivative. Peak 1: kanamycin-OPA derivative, with kanamycin in poultry feeds at levels of 10 mg/g, 40 mg/g, 80 mg/g, and 200 mg/g.

OPA is a widely used derivatization reagent that introduces chromophores in HPLC methods using UV or fluorescence detection. A typical example is a pre-column derivatization of kanamycin with OPA in animal feeds; the reaction scheme is presented in Table 4 [35]. Oasis MCX SPE was used for cleanup. Chromatographic separation was implemented on a XTerra C18 column (250 × 4.6 mm, 5 μm). LOD was 5 g/ton in animal feeds with fluorescence measurement at excitation wavelength of 230 nm and emission wavelength of 389 nm. The HPLC-FL Chromatogram of the kanamycin-OPA pre-column derivative is shown in Figure 7 [35].

**Figure 8:**
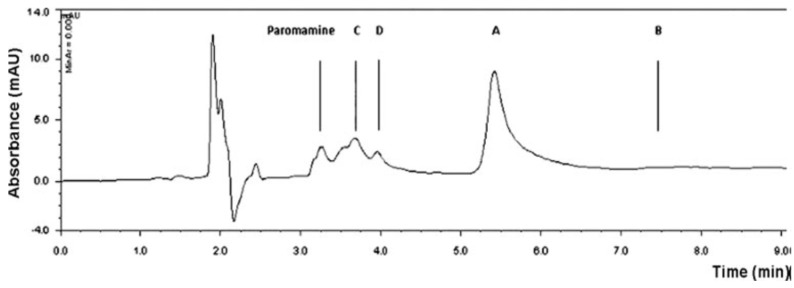
The HPLC-UV chromatogram of kanamycin A borate complexation. Chromatogram obtained after injection of kanamycin A solution (1 g/L) spiked with kanamycins B, C, and D, and paromamine (0.1 g/L each).

Although the pre-column derivatization methods can avoid using ion pair reagent (IPR), IPR is still needed under certain conditions. The derivatization of kanamycin using borate complexation is an example of this [9]; with reaction scheme is shown in Table 4. The HPLC-UV chromatogram of the kanamycin A-borate derivative is shown in Figure 8 [9]. Borate ion was obtained by dissolving borax in water. After borate complexation formation, the derivatives were analyzed with a XBridge C18 column (250 × 4.6 mm, 5 μm), using sodium octanesulphonate as IPR, and with UV detection at 205 nm. Baseline separation from kanamycins B, C, and D were achieved.

#### 3.1.2. Post-Column Derivatization

Post-column derivatization requires more complicated instruments [47] and is confined by reaction time and the solvent system. However, the chemical reaction does not need to be complete since it is repeatable, and long-term stability of the derivative is not a concern [47].

**Figure 9:**
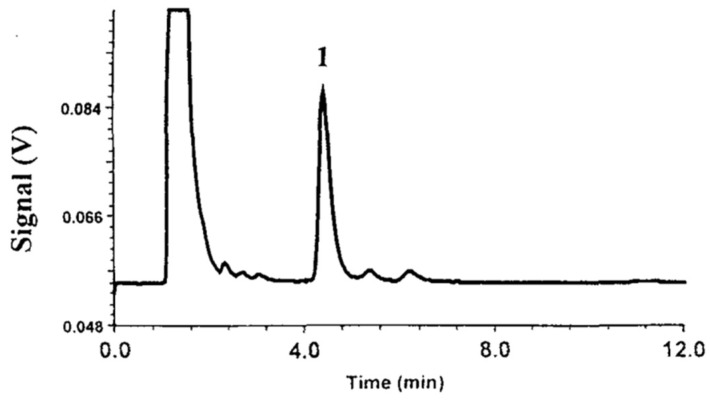
The HPLC-FL chromatogram of the kanamycin-OPA post-column derivative. Peak 1: kanamycin-OPA derivative, with kanamycin in swine feed at a level of 120 mg/kg.

OPA could be used as both pre-column [35] and post-column [19] derivatization agent. Post-column derivatization of kanamycin using OPA was achieved after RPLC with a C8 TSK ODS 120T (150 × 4.6 mm, 5 μm) or Hypersil ODS column (150 × 3.2 mm, 5 μm). Both columns led to good results. The HPLC-FL chromatogram of the kanamycin-OPA post-column derivative is shown in Figure 9 [19]. LOD was 0.2 mg/L in pig feeds, detected with fluorescence measurement at excitation wavelength of 355 nm and emission wavelength of 415 nm.

### 3.2. ELSD and PED-Ion Pair Liquid Chromatography

In ion-pair liquid chromatography (IPLC) methods, the ion pairing reagent (IPR) is used as a mobile phase additive, which interacts with the RPLC stationary phase [47] and allows separating of the ionic and highly polar compounds on RP-HPLC columns. Alkyl sulfonates compounds like octanesulfonate could be used as IPR [10]. Meanwhile, volatile TFA and heptafluorobutyric acid (HFBA) [50,52] could also be used as IPR when coupled with MS detection. Since the high potency of IPR (>20 mM) is harmful to the column packing material, it is ideal to minimize the potency so as to achieve appropriate retention and peak shape [47]. 

In the IPLC method, an extra buffer system is required to maintain a stable pH of the mobile phase [47]. Ammonium acetate and phosphate are the most frequently used buffer solutions. Phosphate buffer is compatible with UV but not with an MS or ELSD detector. Meanwhile, ammonium acetate buffer is incompatible with UV but compatible with an MS detector [47].

#### 3.2.1. Evaporative Light Scattering Detection (ELSD)

ELSD is increasingly being applied in IPLC for compounds without chromophores, because it eliminates the necessity of derivatization [50]. For HPLC applications in the analysis of kanamycin with ELSD detection, refer to Table 2. Some applications of the IPLC-ELSD methods are discussed hereinafter.

The separation of kanamycins A, B, and sulfate were validated through a novel IPLC-ELSD method without the derivatization step. Chromatographic separations were carried out with a Spherisorb ODS-2C18 column (250 × 4.6 mm, 5 μm) using 11.6 mM HFBA as IPR. The LODs were 0.20 μg/mL for kanamycin A, 1.4 μg/mL for kanamycin B and 2.3 μg/mL for kanamycin sulfates [50]. Another example of the IPLC-ELSD method was determination of kanamycin B and tobramycin impurities with HFBA as IPR. Kanamycin was separated on an Agilent SB-Aq C18 column (150 × 4.6 mm, 5 μm) after sample extraction on a weak acidic cation-exchange resin CD180 [52].

HILIC is a very important alternative approach for the separation of kanamycin. A new HILIC-coupled ELSD method was applied for kanamycin detection. In this research, a HILIC column Click TE-Cys (150 × 4.6 mm, 5 μm) was applied for selective separation of kanamycin. High buffer potency (≥50 mM) and low pH (2.7 or 3.0) are required for the mobile phase to improve peak shape and selectivity [51].

#### 3.2.2. Pulsed Electrochemical Detection (PED)

HPLC together with pulsed electrochemical detector (PED) has been adopted in US Pharmacopoeia [50]. Analysis of kanamycin A and its related substances using IPLC coupled with PED has been reported [10,53]. For IPLC-ELSD applications in the analysis of kanamycin, refer to Table 2.

In Adams’ work, octanesulfonate was selected as the IPR. To improve the sensitivity of PED detection, 0.5 M NaOH was added in the post-column effluent to adjust the pH to 13. The packing materials of column PLRP-S (250 × 4.6 mm, 8 μm) was poly (styrene-divinylbenzene). Eight components including kanamycin B and D were separated, and the method was applied to commercial samples [53]. 

Manyanga improved Adams’ work [53] and applied the method to silica-based columns Platinum EPS (150 × 4.6 mm, 3 μm). The amount of salt in the mobile phase was reduced to improve stability, with the use of IPR of octanesulfonate remaining [10]. This method indicated better selectivity and sensitivity.

Nevertheless, the PED method has some disadvantages [54]. First, experience is important for repeatable quantitative results. Second, long equilibration time is required after washing of the electrodes of the electrochemical cell. Therefore, the PED method demands further improvement.

### 3.3. Liquid Chromatography-Mass Spectrometry

**Figure 10:**
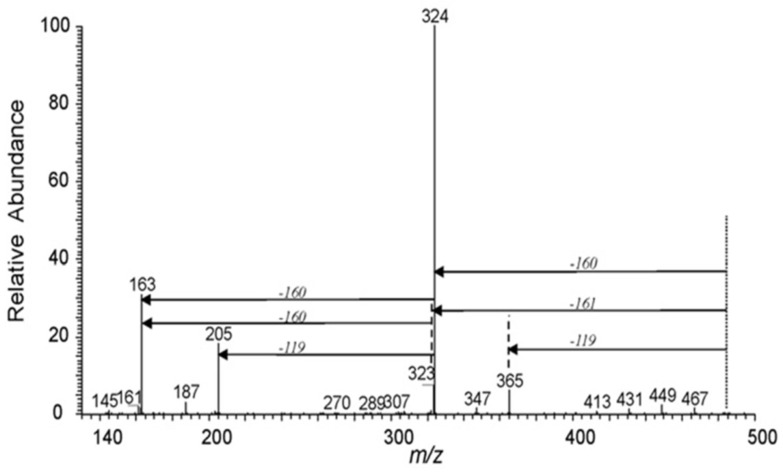
MS/MS spectra of [M + H]^+^ ions of kanamycin B at *m*/*z* 484.

**Figure 11:**
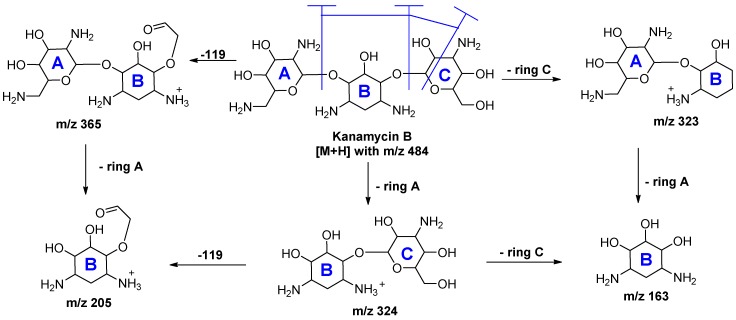
Summary of the fragmentation pathway of kanamycin B reference substances.

LC-MS/MS is a common analytical method in antibiotics residue analysis [33]. Applications of MS with RPLC, IPLC, HILIC or ZIC-HILIC in the analysis of kanamycin are discussed below; refer to Table 3. Mass spectral acquisition was performed in positive-ion mode by applying multiple reactions monitoring (MRM) using electrospray ionization (ESI) or atmospheric pressure chemical ionization (APCI) to detect kanamycin in this review. Kanamycin B produced [M + H]^+^ ions at *m*/*z* 484, which is the precursor ion (Q1). The most abundant product ion (Q3) from the fragmentation was at *m*/*z* 324, and the relatively abundant product ions were *m*/*z* 205 and *m*/*z* 163. The three transition Q3 fragments of kanamycin were 163 for KANA1, and 324 or 205 for KANA2, respectively. The MS/MS spectra of kanamycin B was shown in Figure 10, and the fragmentation pathway of kanamycin B was shown in Figure 11 [55].

#### 3.3.1. IPLC-MS/MS

The IPLC with MS/MS is a powerful tool commonly used in the separation of aminoglycosides [56,57]. The widely used IPRs in kanamycin IPLC-MS-MS analysis were HFBA [17,21,42], TCA [15] and Nonafluoropentanoic acid (NFPA) [20].

In a recent example, kanamycin along with other 12 aminoglycoside antibiotics (AGs) was determined in muscle, kidney, liver, honey, and milk [33]. Volatile HFBA was used as IPR, which was compatible with mass spectrometry and could cause strong retention on the reversed-phase column. Separation was performed using Capcell Pak C18 UG120 column (150 × 2.0 mm, 5 μm). Tobramycin was used as an internal standard (IS). Another rapid qualitative determination of 9 AGs including kanamycin in bovine matrix was realized by IPLC-MS/MS on a Waters BEH C18 (50 × 2.1 mm, 1.7 μm) column, using HFBA as IPR and tobramycin as IS. Since the column material particles were only 1.7 μm ID, the analysis time was shortened to 2.4 min [42]. In another multi-residue study, kanamycin together with 35 other antibiotics were detected in chicken meat on a Betasil phenyl hexyl column (50 × 2.1 mm, 3 μm) [21]. HFBA was chosen as an optimal IPR with minor or no ion suppression effect. For kanamycin detection, LOQ was 25 μg/kg, the decision limit CCα was 121 μg/kg, and detection capability CCβ was 143 μg/kg. Another example was the determination of kanamycin and amikacin in serum using IPLC-MS [17]. IPLC separation was achieved through a water-methanol gradient, containing 0.05% HFBA as IPR, on a Thermo ScientificTM HyPURITYTM C18 column (5.0 × 2.1 mm, 3 μm). Apramycin was used as IS solution [17]. 

Kanamycin, gentamicin and apramycin were quantified in rat plasma by Cheng et al. [15]. In this research, TCA acted as both a protein precipitator and an IPR, which only existed in the sample but not in the mobile phase; yet the system yielded better sensitivity. The absence of TCA in the mobile phase could reduce the contamination of ion source and result in good reproducibility [15]. The retention of AGs was improved on the Phenomenex Synergi C12 Max-RP column (50 × 2.0 mm, 4 μm), using tobramycin as the internal standard.

In a multi-residue analysis, kanamycin and nine other AGs were detected in bovine milk and bovine, swine and poultry muscle using a Waters X-Terra C18 column (100 × 2.1 mm, 3.5 μm) [20]. NFPA was used as IPR in the mobile phase, which improved kanamycin retention in the C18 column and improved its ionization, enhancing the MS/MS signal. Monitoring and screening was performed by LC-QTOF-MS and then confirmed by the LC-MS/MS method. LOQs for kanamycin were 37.5 ng/g for milk and 25 ng/g for muscle. The LODs for kanamycin was 15 ng/g in milk and muscle [20].

#### 3.3.2. HILIC-MS/MS

HILIC shows a similar separation to normal phase liquid chromatography (NPLC), but it can also use water and volatile buffering solution as the mobile phases of RPLC, which are compatible with MS. Therefore, this technique can be applied to separate strong polar and hydrophilic chemical compounds [47]. 

Kanamycin is extremely hydrophilic because it has many amino and hydroxyl groups, so it has good solubility in the aqueous mobile phases of HILIC [58]. There is no need to use IPR in the mobile phase of HILIC, so it will cause less ion suppression and is fully compatible with MS systems. HILIC can provide higher sensitivity because the organic solvent-rich mobile phase is more volatile and can enhance desolvation and ionization efficiency of the ESI source [47].

#### 3.3.3. ZIC-HILIC-MS/MS Method

In recent years, HILIC-coupled mass spectrometry has been successfully applied to the separation of AGs. The application of HILIC to quantify kanamycin and other 5 AGs in human serum was reported [37], with a zwitterionic ZIC-HILIC column (100 × 2.1 mm). LOQ of the method was 100 ng/mL for kanamycin [37]. 

Another application was reported in kidney and muscle tissues using a ZIC-HILIC column (100 × 2.1 mm, 5 μm) [44]. The LOQ of kanamycin was low—50 ng/g. It was observed that the high sorption affinity of kanamycin to polar surfaces required only polypropylene during sample preparation and storage, thus glass was avoided [44]. 

Kanamycin together with six other AGs was determined in veal muscle, and a ZIC-HILIC column (50 × 2.1 mm, 5 μm) was applied [22]. The ZIC-HILIC column (50 × 2.1 mm, 3.5 μm) was also used to determine kanamycin in honey, milk and pork samples [27]. 

Kumar et al. compared six kinds of HILIC stationary phases, including bare silica (anionic), amino phenol (cationic), amide (neutral), and zwitter ionic (ZIC) materials [39]. They concluded that the ZIC phase offered the best result, which might be attributed to the ZIC phase providing interaction with both the electropositive amino and the electronegative hydroxyl. The zwitterionic ZIC-HILIC column (150 × 2.1 mm, 3.5 μm) was used to determine Kanamycin A disulphate dihydrate in honey matrix. Amikacin was selected as the internal standard. The linearity range was 70–2000 μg/L. LOD and LOQ were 8 μg/L and 27 μg/L, respectively [39]. The year after that research, the above-mentioned method was improved and applied to the honey and kidney sample analysis for kanamycin, and validated according to Commission Decision 2002/657/EC. The CCα were 50 μg/kg for honey and 2733 μg/kg for kidney. LOQs were 41 μg/kg for honey and 85 μg/kg for kidney, respectively. The linearity was narrowed down to 70–495 μg/kg for honey and 200–4375 μg/kg for kidney [40]. 

In another similar study, kanamycin was detected in muscle, kidney (cattle and pig) and cow’s milk using ZIC-HILIC column (100 × 2.1 mm, 5 μm) [29], and the internal standard tobramycin was used. The CCα ranges from 118 μg/kg to 2829 μg/kg, and the CCβ range from 153 μg/kg to 3401 μg/kg [29]. 

The usage of a new ZIC-HILIC column Obelisc R (100 × 2.1 mm, 5 μm) was also reported when detecting kanamycin in honey, milk and liver [41]. Obelisc R is a mixed-mode zwitterionic-type LiSC stationary phase, which has a similar structure to ZIC-HILIC column. However, Obelisc R is better than ZIC-HILIC because it has better sensitivity for AGs. The CCα ranges from 3 μg/kg to 793 μg/kg, and CCβ ranges from 5 μg/kg to 881 μg/kg [41].

#### 3.3.4. Other HILIC-MS/MS Methods

The HILIC column CAPCELL PAK ST (150 × 2.0 mm, 4 μm) was applied in separation of 15 AGs residues including kanamycin in animal tissues, milk and eggs [43]. Measurement was carried out through a Thermo electron TSQ Quantum MS. The CCβ of kanamycin ranges from 17.4 μg/kg to 21.9 μg/kg, which was lower than the MRL defined by EU, USA and other countries [43]. 

In another analysis, kanamycin was separated through an Atlantis HILIC column (150 × 2.1 mm, 3 μm) [14], using apramycin as the internal standard. The calibration range was 100–2500 ng/mL for kanamycin in human plasma [14].

A new Click TE-Cys HILIC column (150 × 3 mm, 3 μm) was used to separate kanamycin in milk sample [30]. The LOD and LOQ were 6.1 μg/kg and 19.4 μg/kg, respectively, and the calibration range was 40 ng/mL to 4000 ng/mL [30]. 

The Phenomenex Kinetex HILIC column (100 × 2.1 mm, 1.7 μm) was applied to analyze kanamycin residues in different kinds of milk [28]. The LOD and LOQ were 13.6 μg/kg and 45.5 μg/kg, respectively, and the calibration range was 45.5 μg/k to 250 μg/kg kanamycin in milk [28]. 

Waters HSS T3 column (50 × 2.1 mm, 1.8 μm) was used to analyze kanamycin in serum, gentamicin as IS solution. The LOD and LOQ were 0.5 μg/mL and 2.5 μg/mL, respectively [16]. The LOD and LOQ were further expanded to 0.3 μg/mL and 5.0 μg/mL, respectively, and tested in dried blood spots (DBSs) samples in another study [18].

## 4. Conclusions

The extraction and clean-up methods play a very important role in the analysis of kanamycin. A series of information on methodologies for extraction and clean-up of kanamycin have been published. The extraction and clean-up methods for kanamycin have been applied to a variety of matrices, including animal feeds, liver and kidney tissues, and serum, among others. When the sample contains protein, as milk and serum, protein precipitation is an initial and key step. After protein precipitation, liquid-liquid extraction can be performed to remove fats by using hexane. SPE can be used to remove salts that might affect the ionization of the MS detector.

Much progress has been achieved in kanamycin detection. However, numerous problems still exist and need to be addressed. The UV and fluorescence derivatization methods are time consuming, and the reaction by-products often cause difficulties in quantitation. Therefore, simpler and direct detection methods are preferred, such as PED [10] and ELSD [50,51,52]. Nevertheless, ELSD is less sensitive than PED, needs to use volatile additives, and does not display a direct linear relation with the amount injected [10]. Some are semi-quantitative determination methods. LC-MS/MS methods can ensure good sensitivity and separation ability to detect kanamycin in animal-origin food [21,30]. However, the required instruments are not commonly available in many laboratories owing to their high cost. The IPLC is also suitable for MS-MS detector, while the IPR must be volatile and compatible with MS detector with low ionization suppression. Besides IPLC, HILIC is fully compatible with MS systems and free from IPR in the mobile phase. Meanwhile, the HILIC method can achieve lower detection limits [47]. Therefore, the HILIC-MS-MS offers further direction. Moreover, the MRLs of kanamycin residues defined by the EU Commission Decision is still not quite comprehensive, such as the absence of honey; thus, more sample materials needed to be included. We hope that this paper provides some help for kanamycin detection.

## Figures and Tables

**Table 1:** HPLC applications in the analysis of kanamycin A with UV and fluorescence detection.

Detection	Matrix	Compound of Interest	Derivatization Agent and Condition	Extraction and Cleanup Methods	HPLC Type	Column Type and Temperature	Mobile Phases	Detector Wave Length	Recovery (%)	LOD	LOQ	Refs
**UV**	solvent	KANA A	disodium tetraborate, added in mobile phase	-	IPLC	Reversed-phase column, XBridge C18 (250 × 4.6 mm, 5 μm), 50 °C	0.1 M disodium tetraborate (pH 9.0)-water (20:80, *v*/*v*) with 0.5 g/L sodium octanesulphonate, isocratic.	205 nm	-	38 mg/L	128 mg/L	[9]
**UV**	soil	KANA A	CNBF, PH 9.0, 10 min, 70 °C	**SPE**, AccuBOND ODS-C18	RPLC	Reversed-phase column, kromasil C18 (250 × 4.6 mm, 5 μm).	methanol-0.1% TFA in water, gradient	245 nm	72.3–92.5	0.006 mg/kg	0.01 mg/kg	[31]
**UV**	solvent	KANA A	PIC, 10 min, 70 °C	-	RPLC	Reversed-phase column, Phenomenex C18 (250 × 4.6 mm, 5 μm)	ACN-1% tris buffer (40:60, *v*/*v*) pH adjusted to 6.5 with 1 N sulfuric acid, isocratic	242 nm	92–98	0.597 μg/mL	1.021 μg/mL	[48]
**UV**	human plasma	KANA A	NITC in pyridine, 70 °C	**Protein Precipitation** with can	RPLC	Reversed-phase column, LichrocartPurospher STAR C18 (55 × 4 mm, 3 μm)	water-methanol (33:67, *v*/*v*), isocratic	230 nm	95.9–100.8	0.3 μg/mL	1.2 μg/mL	[13]
**FL**	animal feeds	KANA A	OPA-ME	**SPE**, Oasis MCX cartridge (3 cc, 60 mg)	RPLC	Reversed-phase column, XTerra^TM^C18 (250 × 4.6 mm, 5 μm)	ammonium acetate solution-ACN (50:50, *v*/*v*), isocratic	Ex:230 nm; Em: 389 nm	98.4–106	5 g/ton	10 g/ton	[35]
**FL**	swine tissue	KANA A	FMOC-Cl, 15 min, RT	**SPE**, Oasis MCX cartridge (3 cc, 60 mg)	RPLC	Reversed-phase column, Waters symmetry C18 (150 × 3.9 mm, 5 μm)	ACN-water, gradient	Ex: 260 nm; Em: 315 nm	80.7–91.3	muscle: 0.03 mg/kg; liver: 0.06 mg/kg; kidney: 0.18 mg/kg	muscle: 0.1 mg/kg; liver: 0.2 mg/kg; kidney: 0.6 mg/kg.	[36]
**FL**	human plasma	KANA A	FMOC-Cl, 30 min, 25 °C	**Protein Precipitation** with ACN	RPLC	Reversed-phase column, Eclipse XDB C8 (150 × 4.6 mm, 5 μm), 25 °C	70% ACN, isocratic	Ex: 268 nm; Em: 318 nm	92.3–100.8	0.01 μg/mL	0.05 μg/mL	[12]
**FL**	wastewater and soil	KANA A	FMOC-Cl, 15 min, RT	**DLLME-SFO**, extraction solvent: dodecanol. dispersive solvent: ethanol	RPLC	Reversed-phase column, Diamonsil C18 (250 × 4.6 mm, 5 μm), 40 °C	ACN-water (84:16 *v*/*v*), isocratic	Ex: 265 nm; Em: 315 nm	Wastewater: 91.3–102.7; soil: 90.3–107.7	0.012 ng/mL	0.05 ng/mL	[23]
**FL**	pig feeds	KANA A	OPA-ME	**Protein Precipitation** with 0.1 M HCl	RPLC	Reversed-phase column, Eluent B: TSK ODS 120T (150 × 4.6 mm, 5 μm). Eluent C: Hypersil ODS for (150 × 3.2 mm, 5 μm), RT	Eluent B: THF-15 mM sodium sulphate, 3:97(*v*/*v*). Eluent C: 10 mM acetic acid-10 mM pentane sulphonate, 1.5:98.5 (*v*/*v*)	Ex: 355 nm; Em: 415 nm	89.4–92.8	0.2 mg/L	0.4 mg/L	[19]

FL: Fluorescence, KANA: Kanamycin, ACN: Acetonitrile.

**Table 2:** HPLC applications in the analysis of kanamycin with ELSD and PED.

Detection	Matrix	Compound of Interest	Extraction and Cleanup Methods	HPLC Type	Column Type and Temperature	Mobile Phases & IPR	Recovery (%)	LOD	LOQ	Refs
**ELSD**	solution	Kanamycin A, B and Sulfates	-	reversed-phase IPLC	Reversed-phase column, Spherisorb ODS-2 C18 (250 × 4.6 mm, 5 μm), RT	water-ACN (60:40, *v*/*v*), 11.6 mM HFBA, isocratic	Kanamycin A: 95–103, Kanamycin B: 95–105	Kanamycin A: 0.2 μg/mL, Kanamycin B: 1.4 μg/mL.	Kanamycin A: 0.6 μg/mL, Kanamycin B: 4 μg/mL	[50]
**ELSD**	solution	Kanamycin sulfate	-	HILIC	Mixed-mode column, Click TE-Cys (150 × 4.6 mm, 5 μm), 30 °C	ammonium formate aqueous solution (A)-ACN (B)-water(C), gradient	-	-	-	[51]
**ELSD**	fermentation broth	Kanamycin B	CD180 resin column	reversed-phase IPLC	Reversed-phase column, Agilent SB-Aq C18, RT	water-ACN (65:35, *v*/*v*), 11.6 mM HFBA, isocratic	93–96	-	0.05 mg/mL	[52]
**PED**	solution	Kanamycin	-	IPLC	Reversed-phase column, Platinum EPS (150 × 4.6 mm, 3 μm), 45 °C	**MPA**: sodium sulphate (5.0 g/L), sodium octanesulphonate (0.5 g/L) and 0.2M phosphate buffer pH 3.0 (50.0 mL/L) **MPB**: sodium sulphate (15 g/L), sodium octanesulphonate (0.5 g/L) and 0.2M phosphate buffer pH 3.0 (50.0 mL/L) Octanesulfonate as IPR, gradient	-	1.7 ng	5 ng	[10]
**PED**	solution	Kanamycin B,D	-	IPLC	Reversed-phase column, PLRP-S column packed with polystyrene-divinylbenzene, (250 × 4.6 mm, 8 μm), 45 °C	**MPA**: sodium sulphate (20 g/L), sodium octanesulphonate (1.3 g/L) and 0.2M phosphate buffer pH 3.0 (50.0 mL/L) **MPB**: sodium sulphate (60 g/L), sodium octanesulphonate (1.3 g/L) and 0.2M phosphate buffer pH 3.0 (50.0 mL/L). Octanesulfonate as IPR, gradient	-	Kanamycin D: 3 ng, Kanamycin B: 5 ng	Kanamycin D: 10 ng, Kanamycin B: 15 ng	[53]

ACN: Acetonitrile.

**Table 3:** HPLC applications in the analysis of kanamycin with MS detection.

Detection	Matrix	Compound of Interest	Extraction and Clean-up Methods	HPLC Type	Column Type and Temperature	Mobile Phases	Recovery (%)	LOD	LOQ	Refs
**LC-MS/MS**	rat plasma	kanamycin	**Protein Precipitation** with TCA added in sample	IPLC	Reversed-phase column, PhenomenexSynergi C12 Max-RP (50 × 2.0 mm, 4 μm)	0.1% FA in water-0.1% FA in ACN, gradient	-	-	20 ng/mL	[15]
**UPLC-MS/MS TQD**	bovine kidney, liver, muscle	kanamycin sulfate	**SPE**, Disposable pipet extraction (DPX), 5 mL tips containing 50 mg WCX sorbent	IPLC	Reversed-phase column, Waters BEH C18 (50 × 2.1 mm, 1.7 μm)	20 mM HFBA in 5% ACN in H_2_O-20 mM HFBA in ACN, gradient, 3min. Tobramycin as IS	84–92	-	0.005 μg/g	[42]
**LC-MS/MS**	muscle, kidney, liver, honey and milk	kanamycin	**SPE**, two-coupled Oasis HLB columns (3 mL/60 mg)	IPLC	Reversed-phase column, Capcell Pak C18 UG120 (150 × 2.0 mm, 5 μm), 30 °C	20 mM HFBA in 5% ACN-20 mM HFBA in 50% ACN, gradient,10 min. 500 ng/mL Tobramycin as IS	71–104	CCα (μg/kg) 49.5 for muscle, 48.9 for liver, 49.1 for kidney, 9.8 for honey, 121.5 for milk	CCβ (μg/kg) 60.2 for muscle, 58.4 for liver, 59.4 for kidney, 12.4 for honey, 146.4 for milk.	[33]
**LC-MS/MS TQD**	chicken meat	kanamycin	**Protein Precipitation** with ACN: 2% TCA (45:55, *v*/*v*). On-line clean-up using column: Thermo Cyclone P (50 × 0.5 mm, 60 μm)	IPLC	Mixed-mode column, Thermo Betasil phenyl hexyl (50 × 2.1 mm,3 μm), RT	1 mM HFBA and 0.5% FA in water-0.5% FA in ACN/methanol (1:1, *v*/*v*), gradient, 19 min	109–120	LOD: 10.0 μg/kg CCα: 121.3 μg/kg	LOQ: 25.0 μg/kg CCβ: 142.5 μg/kg	[21]
**LC–MS/MS TQD**	human serum	kanamycin	**Protein Precipitation** with TCA added in sample	IPLC	Reversed-phase column, Thermo Scientific^TM^HyPURITY^TM^C18 (5.0 × 2.1 mm, 3 μm)	water/methanol, containing 0.05% HFBA, gradient. Apramycin as IS	93.9–98.4	-	100 ng/mL	[17]
**LC-MS-TOF**	bovine milk & swine, poultry muscle	kanamycin	**Protein Precipitation** with TCA added in sample, then supernatant go through a tube containing bulk C18 resin	IPLC	Reversed-phase column, Waters X-Terras C18 (100 × 2.1 mm, 3.5 μm)	10 mM NFPA in H_2_O-10 mM NFPA in ACN, gradient	milk: 92, muscle: 36.8–67	15 ng/g for milk and muscle	37.5 ng/g for milk, 25 ng/g for muscle	[20]
**LC-MS/MS**	muscle, liver, kidney, milk, egg	kanamycin	**SPE**, CBA cartridge (10 CC, 500 mg)	HILIC	HILIC column, CAPCELL PAK ST (150 × 2.0 mm, 4 μm), 30 °C	ACN-0.1%TFA, gradient	75–98	CCα (μg/kg) 8.1 for muscle, 8.5 for egg, 10.0 for liver, 11.2 for kidney, 11.5 for milk	CCβ (μg/kg) 17.6 for muscle, 21.9 for egg, 19.1 for liver, 17.4 for kidney, 18.5 for milk	[43]
**LC-MS/MS**	milk	kanamycin	Consecutive **SPE** of Sep-pak tC18 (6 mL/500 mg) and Oasis WCX (6 mL/500 mg), extraction with 3% TCA	HILIC	HILIC column, Click TE-Cys HILIC (150 × 3 mm, 3 μm)	1% FA in H_2_O-1% FA in 80% ACN, both containing 30 mM ammonium formate, gradient, 10 min	69.9–77.9	6.1μg/kg	19.4μg/kg	[30]
**UPLC-MS/MS**	milk sample	Kanamycin acid salt	**SPE**, Supel, MISPE-Aminoglycoside cartridge (3 mL/50 mg)	HILIC	HILIC column, PhenomenexKinetex HILIC (100 × 2.1 mm, 1.7 μm), 35 °C	150 mM ammonium acetate in 1% FA(A)-ACN(B), gradient, 12 min	70–106	13.6 μg/kg	45.5 μg/kg	[28]
**LC-MS/MS**	human plasma	kanamycin	**Protein Precipitation** with acidified methanol using HCl	HILIC	HILIC column, Atlantis HILIC (150 × 2.1 mm, 3 μm), 35 °C	0.1% FA in water-0.1% FA in ACN, gradient, 9.0 min	91.2–93.4	-	1 μg/mL	[14]
**UPLC-MS/MS**	human serum	kanamycin	**Protein Precipitation** with acidified methanol using HCl	HILIC	Reversed-phase column, Waters HSS T3 (50 × 2.1 mm, 1.8 μm), RT	10 mM ammonium formate in 0.1% FA-ACN in 0.1% FA, gradient, 3 min	-	0.5 μg/mL	2.5 μg/mL	[16]
**UPLC-MS/MS**	dried blood spots	kanamycin	**Protein Precipitation** with acidified methanol using HCl	HILIC	Reversed-phase column, Waters Acquity HSS T3 (50 × 2.1 mm, 1.8 μm)	10 mM ammonium formate in 0.1% FA-ACN in 0.1% FA, gradient, 4 min	-	0.3 μg/mL	5.0 μg/mL	[18]
**Quattro Ultima LC-MS/MS**	muscle and kidney	kanamycin	**SPE**, CBX cartridge (500 mg)	ZIC-HILIC	HILIC column, SeQuant ZIC-HILIC (100 × 2.1 mm, 5 μm), 32 °C	1% FA in 150 mM ammonium acetate-ACN, gradient, 19 min	81.1–104	18 ng/g	58 ng/g	[44]
**LC–MS/MS**	muscle, kidney and milk	kanamycin	**SPE**, Chromabond HR-X cartridge (6 mL/500 mg)	ZIC-HILIC	HILIC column, SeQuant ZIC-HILIC (100 × 2.1 mm, 5 μm), 30 °C	1% FA with 200 mM ammonium acetate in 5% ACN-ACN, gradient, 16 min	95–102	CCα (μg/kg) 118 for muscle, 2829 for kidney, 172 for milk	CCβ (μg/kg) 153 for muscle, 3401 for kidney, 215 for milk	[29]
**Quattro Ultima UPLC-MS/MS**	honey samples	Kanamycin A disulphatedihydrate	**SPE**, WCX cartridge, Accell Plus CM (6 mL/500 mg)	ZIC-HILIC	HILIC column, SeQuant ZIC-HILIC (150 × 2.1 mm, 3.5 μm), 40 °C	pH 4.5 with 125 mM ammonium formate-0.2% FA in ACN, gradient, 6 min. Amikacin as IS	68–112	8 μg/L	27 μg/L	[39]
**Quattro Premier**	kidney and honey	Kanamycin A disulphatedihydrate	**SPE**, WCX cartridge, Accell Plus CM (6 mL/500 mg)	ZIC-HILIC	HILIC column, SeQuant ZIC-HILIC (150 × 2.1 mm,3.5 μm), 40 °C	PH 4.5 with 175 mM ammonium formate-0.2% FA in ACN, gradient, 6 min. Amikacin as IS	82–105	CCα (μg/kg): 50 for honey, 2733 for kidney	CCβ (μg/kg) 67 for honey, 2965 for kidney. LOQ (μg/kg): 41 for honey, 85 for kidney.	[40]
**UPLC-MS/MS**	honey, milk and liver	kanamycin	**SPE**, Taurus WCX cartridge	ZIC-HILIC	Mixed-mode column, Obelisc R (100 × 2.1 mm, 5 μm), 40 °C	0.1% FA in water-0.1% FA in ACN, gradient, 8.0 min	58–96	LOD (μg/kg): 1 for honey, 1 for milk. CCα: 3 for honey, 172 for milk, 793 for liver	LOQ (μg/kg): 3 for honey, 5 for milk. CCβ: 5 for honey, 175 for milk 881 for liver	[41]
**HILIC-MS/MS**	honey, milk and pork	kanamycin disulfate salt	**SPE**, Supel MISPE-Aminoglycoside cartridge (3 mL/50 mg)	ZIC-HILIC	HILIC column, Zwitterionic HILIC (50 × 2.1 mm, 3.5 μm), 40 °C	175 mmol/L ammonium formate and 0.3% FA-methanol and 0.3% FA, gradient, 8 min	72.8–97	10 μg/kg for honey, 11 μg/kg for milk and pork	34 μg/kg for honey, 36 μg/kg for milk and pork	[27]
**HILIC-MS/MS**	human serum	kanamycin	**SPE**, Oasis MCX cartridge (30 mg)	ZIC-HILIC	HILIC column, SeQuant ZIC-HILIC (100 × 2.1 mm)	A (5/95/0.2, *v*/*v*/*v*) and B (95/5/0.2, *v*/*v*/*v*) each being a mixture of ACN: 2 mM ammonium acetate: FA, gradient	-	-	100 ng/mL	[37]
**LC-MS/MS**	veal muscle	kanamycin disulfate salt	**LLE**, defatting using hexane	ZIC-HILIC	HILIC column, ZIC-HILIC (50 × 2.1 mm, 5 μm)	0.4% formic acid inwater/ACN, gradient, 15 min	-	6 ng/g	-	[22]

**Table 4:** Reaction schemes of kanamycin with different derivatization regents.

Reaction Scheme	Refs
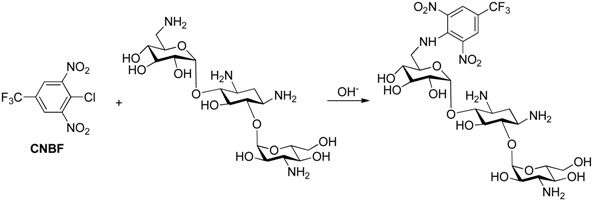	[31]
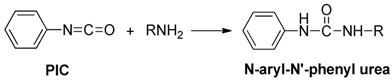	[48]
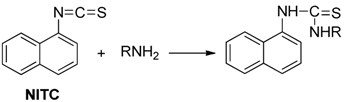	[13]
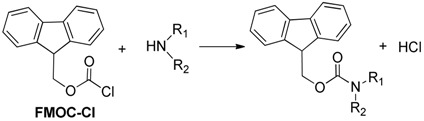	[12]
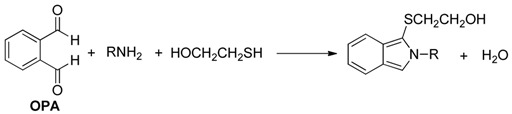	[36]
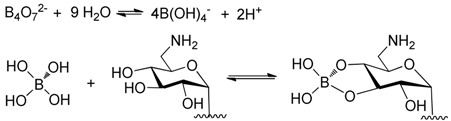	[9]

## Abbreviations

ACN	Acetonitrile
AGs	Aminoglycoside antibiotics
anti-TB drug	anti-Tuberculosis drug
APCI	Atmospheric Pressure Chemical Ionization
CBA	Carboxylic Acid
CBX	Carboxypropyl
CCα	Decision Limit
CCβ	Detection Capability
CNBF	4-chloro-3,5-dinitrobenzotrifluoride
DBSs	Dried Blood Spots
DLLME-SFO	Dispersive Liquid-Liquid Microextraction Based on Solidification of Floating Organic Droplet
DPX	Disposable (or Dispersive) Pipet Extraction
ELSD	Evaporative light scattering detection
ESI	Electrospray ionization
FA	Fomic acid
FL	Fluorescence
FOMC-Cl	9-Fluorenylmethyl chloroformate
HCl	Hydrochloricacid
HFBA	Heptafluorobutyric acid
HILIC	Hydrophilic interaction chromatography
HLB	Hydrophilic-lipophilic balanced
HPLC	High Performance Liquid Chromatography
IPLC	Ion-pair liquid Chromatography
IPR	Ion-pairing agent
IS	Internal standard
KANA	Kanamycin
LC-MS	Liquid chromatography-mass spectrometry methods
LLE	Liquid-liquid extraction
LOD	Limit of detection
LOQ	Limit of quantification
ME	Mercaptoethanol
MIPs	Molecularly imprinted polymers
MPA	Mobile phase A
MPB	Mobile phase B
MRLs	Maximum residue limits
MRM	Multiple reactions monitoring
MS	Mass Spectrometry
NITC	1-Naphthyl isothiocyanate
NFPA	Nonafluoropentanoic acid
NPLC	Normal Phase liquid Chromatography
ODS	Octadecyl silane
OPA	*O*-phthaladehyde
PED	Pulsed Electrochemical Detection
PIC	Phenylisocyanate
Q1	Precursor ion
Q3	Product ion
RPLC	Reverse phase liquid chromatography
SPE	Solid-phase extraction
TCA	Trichloroacetic acid
TFA	Trifluoroacetic acid
UV	Ultraviolet
ZIC-HILIC	Zwitter ionic-hydrophilic interaction chromatography
